# Native and foreign healthcare workers’ knowledge of appropriate use of antibiotics: a prospective pre-post study in Danish nursing homes

**DOI:** 10.1017/S1463423621000025

**Published:** 2021-04-05

**Authors:** Ida Scheel Rasmussen, Matilde Bøgelund Hansen, Tina Marloth, Magnus Arpi, Jens Otto Jarløv, Sif Helene Arnold, Dorthe Mogensen, Jette Nygaard Jensen

**Affiliations:** 1 Department of Clinical Microbiology, Herlev and Gentofte Hospital, University of Copenhagen, Herlev, Denmark; 2 Department of Clinical Microbiology, Herlev and Gentofte Hospital, University of Copenhagen, Herlev, Denmark and at the Department of Public Health, Research Unit for General Practice, University of Copenhagen, Copenhagen, Denmark; 3 Department of Clinical Microbiology, Herlev and Gentofte Hospital, University of Copenhagen, Herlev, Denmark and consultant at Task Force Reducing Hospital Infections, Capital Region of Denmark, Copenhagen, Denmark

**Keywords:** antibiotic, antimicrobial, healthcare workers, nursing home

## Abstract

**Aim:**

The aim was to determine the association between healthcare workers’ (HCWs) country of birth and their knowledge of appropriate use of antibiotics, and whether the association changed after an educational intervention.

**Background:**

Older residents in nursing homes have been recognized to receive excessively antibiotic treatments. HCWs often represent an important link between the older resident and the general practitioner prescribing the antibiotics, thus their knowledge of appropriate use of antibiotics is important.

**Methods:**

This study was conducted as a prospective pre-post study. Totally, 312 HCWs from 7 nursing homes in Denmark were included. For statistical analyses, χ^2^ test and a linear mixed regression model were applied.

**Findings:**

Native HCWs were more likely to have a higher percentage of correct responses to single statements related to knowledge of appropriate use of antibiotics. Native HCWs had a significantly higher knowledge-of-antibiotic score compared to foreign HCWs (−7.53, *P* < 0.01). This association remained significant after adjusting for relevant covariates (−5.64, *P* < 0.01). Native HCWs’ mean change in knowledge-of-antibiotic score after the intervention did not differ from the foreign HCWs’ mean change in knowledge-of-antibiotic score.

**Conclusion:**

Our findings indicate that HCWs born outside Denmark reveal a lower knowledge-of-antibiotic score than HCWs born in Denmark despite comparable educational backgrounds. All participants increased their knowledge from baseline to follow-up. Our findings also indicate that an educational seminar cannot equalize the difference in knowledge between native and foreign HCWs. Studies with larger sample size and a more detailed measurement of cultural identity should investigate this association further.

## Introduction

The effect of antibiotics on bacterial infection is essential to public health. As stated by the World Health Organization, a significant threat to global health is the incapability to treat common infectious diseases due to antibiotic resistance (World Health Organization, 2017). Inappropriate and redundant use of antibiotics contributes to the development of antibiotic-resistant bacteria (Alzoubi *et al*., 2009; Andersson *et al*., 2012). Therefore, it is crucial to reduce unnecessary prophylactic and inappropriate use of antibiotics.

Antibiotics are commonly used medications among the older people in nursing homes (Centers for Disease Control and Prevention, 2018). A point prevalence study from 2017 showed that on a given day almost 5% of nursing home residents in Europe were treated with at least one antimicrobial agent (Ricchizzi *et al*., 2018), and it has been estimated that about half of the antibiotic prescriptions are inappropriate (Nicolle *et al*., 2000). Denmark was the country with the highest percentage of nursing home residents in antibiotic treatment (Ricchizzi *et al*., 2018). Several studies describe the problem of inappropriate antibiotic prescribing, for example, treatment of asymptomatic bacteriuria, unnecessary prophylactic treatment, or unnecessarily prolonged treatment. Inappropriate use of antibiotics in these situations disregard the lack of effect and adverse consequences that inappropriate use of antibiotics has (Daneman *et al*., 2013; Fleming *et al*., 2013; Rhee and Stone, 2014; Dyar *et al*., 2015; van Buul *et al*., 2015).

Large geographical differences in both consumption of antibiotics in primary care and the knowledge of appropriate use of antibiotics exist (Brauer *et al*., 2016; ECDC, 2017; Hansen *et al*., 2020). Variations in access to antibiotics, pathogen characteristics, legislation, healthcare structure, or the care load of the elderly cannot account for all discrepancies in antibiotic use between countries but may be related to local habits and prescribing practice (Deschepper *et al*., 2008; Ricchizzi *et al*., 2018). Research has repeatedly shown that ethnicity comprising cultural dimensions play an important role in illness behavior and consumption of antibiotics (Mangione-Smith *et al*., 2004; Deschepper *et al*., 2008; Gould and Meer, 2008; Gaygısız *et al*., 2017). Deschepper *et al*. (2002) found that religion likewise had an impact on the perception of illness leading to differences in the consumption of antibiotics (Deschepper *et al*., 2002). We wanted to explore if ethnic disparities in knowledge are apparent among healthcare workers (HCWs) in Danish nursing homes or if the ethnic disparities in knowledge are absent when all HCWs are educated, working, and living in the same country.

HCWs in nursing homes are perceived as central to the appropriate use of antibiotics as they are the link between the nursing home resident and the nursing home resident’s general practitioner. General practitioners’ treatment decisions often rely solely on HCWs’ assessment and interpretation. Commonly, the general practitioners prescribe antibiotics, for example, urinary tract infection without seeing the elderly patient in person, and their treatment is initiated due to observations or dipstick testing provided by the healthcare staff; hence, HCWs’ knowledge, awareness, and attitudes toward the use of antibiotics have major importance (McClean *et al*., 2012; Kirsebom *et al*., 2017; Arnold *et al*., 2020).

Several studies have examined the importance of ethnicity on either general practitioners’, the public’s, or patients’ knowledge regarding antibiotics, whereas the importance of ethnicity when investigating HCWs’ knowledge seems to be undiscovered (Gill *et al*., 1997; Corbett *et al*., 2005; McNulty *et al*., 2007). Even though ethnic groups represent a significant number in nursing homes and influence the prescription decision process, no studies have examined ethnic differences and disparities in HCWs’ knowledge of appropriate use of antibiotics. Since the effect of simple educational interventions according to knowledge and beliefs about the use of antibiotics is questionable, it could be of importance to identify if differences exist in HCWs starting point (Forsetlund *et al*., 2011; Pettersson *et al*., 2011).

The aim of this study was to compare knowledge of appropriate use of antibiotics and antibiotic resistance (henceforth referred to as knowledge of antibiotics) among native HCWs and foreign HCWs in seven Danish nursing homes. Furthermore, this study assesses whether native HCWs and foreign HCWs responded differently to an educational seminar about antibiotics and antibiotic resistance.

## Methods

### Study design and participants

This study was conducted as a prospective pre-post study. Participants were recruited from seven nursing homes in two municipalities in the Capital Region of Denmark. All HCWs employed in the seven nursing homes were invited to participate in the study. The HCWs were represented by varying duration and levels of education: nurses (short-cycle higher education), healthcare assistants (longer vocational education), healthcare helpers (short vocational education), domestic workers (often short vocational education), and unskilled workers (basic schooling). In total, 469 HCWs were invited to participate in the study.

### Survey instruments and data collection procedures

We used a structured self-administered questionnaire to assess the knowledge of antibiotics 1 month before and 2 months after the intervention, respectively. Baseline and follow-up questionnaires were identical except for the addition of questions regarding demographic information in the baseline questionnaire. The HCWs’ ethnicity was specified with an open question asking about the participants’ country of birth. Participants born in Denmark were defined as natives, whereas participants born outside Denmark were defined as foreigners. The questionnaire also covered questions about the HCWs’ educational background, work experience in a nursing home, and work hours/work schedule.

Fourteen statements focusing on bacteria, virus, antibiotics, and antibiotic resistance were used to evaluate the HCWs’ knowledge of antibiotics at baseline and follow-up. Statements evaluating the knowledge of antibiotics were rated on a scale with the response alternatives ‘agree’, ‘disagree’, and ‘do not know’. All statements are presented in Table 2. The questionnaires were developed by the research group by adapting earlier developed questionnaires from other surveys examining knowledge of antibiotics (André *et al*., 2010; World Health Organization, 2015). The two following statements were developed by the research group: ‘*The majority of mild infections get better on their own*’ and ‘*Resistance can eventually lead to incurable infections*’. The final questionnaires were face-validated by experts in clinical microbiology and subsequently appraised and pretested on 12 healthcare professionals. A combined knowledge-of-antibiotic score was calculated as a continuous variable by percentages of correct responses related to answered statements, ranging from 0 to 100% with 100% indicating the most favorable level of ‘knowledge of antibiotics’ and 0% the least favorable.

A member of the research group provided the surveys to one local leader in each nursing home who distributed the surveys to all HCWs. The HCWs completed the questionnaire and subsequently placed it in a sealed box to achieve anonymity. The HCWs were encouraged to fill the questionnaire alone without interruption.

### Intervention: Educational seminar

Two infection control nurses from the two participating municipalities provided the research group with insightful knowledge about issues that should be addressed at the seminar. Furthermore, the baseline questionnaire showed the research group where there was a lack of knowledge among the HCWs, and this was incorporated at the seminars.

All HCWs at the participating nursing homes were invited to participate in a 2-hour on-off seminar. The seminar was structured into three parts: the first part provided general knowledge on bacteria, virus, antibiotics, and antibiotic resistance. The second part outlined the risk factors of urinary tract infection among residents at nursing homes. The third part provided recommendations on prevention and treatment of urinary tract infections at nursing homes. The seminar was always held by a researcher, a medical doctor, and an infection control nurse. This study evaluates the first part of the seminar about bacteria, virus, antibiotics, and antibiotic resistance.

### Statistical analyses

Descriptive and inferential statistics were performed with SAS 9.4 & SPSS 25. We examined the distribution of participants according to relevant demographic characteristics and ethnicity. Frequencies were compared with the use of the χ^2^ test, while continuous variables were compared with the use of Student’s t-test. In all analyses, statistical significance was determined by *P* < 0.05.

Responses to single statements at baseline were compared between groups using percentages of correct responses on each statement. χ^2^ tests were used for each statement to verify differences in correct responses between native HCWs and foreign HCWs.

#### Change in knowledge-of-antibiotic score after educational seminar

Linear mixed model regression analysis was used to estimate the strength of the association between knowledge of antibiotics and ethnicity. The advantage of using a linear mixed mode was that the effect of nursing home clustering was taken into account. Observations with missing data on more than two statements were excluded from the dataset. We included ethnicity, intervention (pretest vs. posttest), and an interaction term between ethnicity and intervention in the model to assess differences in knowledge of antibiotics between ethnic groups prior to and after the educational seminar. Furthermore, we adjusted for work experience and educational background. Place of employment was included as a random effect to adjust for the possible sampling variation due to different nursing homes. Directed acyclic graph was used to identify which measured variables we should adjust for (Shrier and Platt, 2008). Change in knowledge of antibiotics was conducted according to the intention-to-treat concept; therefore, all observations were included regardless of attendance in the seminar.

### Ethical considerations

According to Danish legislation, no ethical approval is needed for questionnaire-based studies (National Research Ethics Committees, 2013). The participants were informed that participation was voluntary. The participants were assured that their responses would be treated confidentially, and any personal information that could identify them would be removed or changed before results were published.

## Results

### Baseline characteristics

Totally, 332 completed the baseline survey, resulting in a response rate of 71%. Due to missing information on origin, one participant was excluded. Nineteen participants were excluded from the dataset because they responded to too few statements (<12 of 14). The final analyses of baseline knowledge of antibiotics were performed on 312 participants representing 39 different birth countries. Characteristics of study participants are displayed in Table 1. The majority was born in Denmark, while 60 of the participants (19%) were born in another country than Denmark. The mean age of the participants was 49 ± 11 years. The majority of the respondents was women (96%), educated healthcare helpers (49.4%), and worked fixed days shifts (56.8%). Overall, the participants had 16.1 ± 10.5 years of work experience in a nursing home. Significant differences between foreign participants and native participants were found in the variable work experience (Table 1).

**Table 1.** Demographic characteristics of healthcare workers (HCWs), baseline

Characteristics	Native HCW *n* = 252, 80.8%	Foreign HCW *n* = 60, 19.2%	Total *n* = 312, 100%	*p* ^†^
Gender				0.3
Female	243 (81.3)	56 (18)	299	
Male	9 (69.2)	4 (30.8)	13	
Missing			0	
Age				0.1
Years (±SD)	50.2 (±11)	47.5 (±10.4)	49.6 (±10.9)	
Missing			7	
Educational background				0.06
Nurse	*		19	
Healthcare assistants	85 (80)	21 (20)	106	
Healthcare helpers	177 (76)	37 (23)	154	
Unskilled workers	*		<5	
Domestic workers	*		<5	
Other^○^	*		25	
Missing			1	
Work hours				0.2
Fixed day shifts	146 (83.4)	29 (16.6)	175	
Fixed evening shifts	68 (77.3)	20 (22.7)	88	
Fixed night shifts	23 (79.3)	6 (20.7)	29	
Flexible schedule	13 (81.3)	3 (18.8)	16	
Missing			4	
Work experience in a nursing home				**<0.0001**
Years (±SD)	17.5 (±10.6)	10.2 (±7.7)	16.1 (±10.5)	
Missing			5	

Bold numbers indicate p-values that are significant on the 95% confidence limit.

○ Kitchen assistants, cleaning staff etc.

† Frequencies were compared with the use of the χ^2^ test. Mean values were compared with the use of Student’s t-test

* Less than three observations in one of the groups

#### Single statements

HCWs’ knowledge of antibiotics was examined using 14 statements related to bacteria, virus, antibiotics, and antibiotic resistance. The 14 individual statements are presented in Table 2. In 13 out of 14 statements, higher percentages of native HCWs responded correctly compared to the percentages of foreign HCWs that responded correctly.

**Table 2.** Distribution of correct responses by native and foreign healthcare workers and χ^2^ test.

	% responding correctly		*P*
Statement	Natives	Foreign	
*Bacteria and virus*			
1. Antibiotics are effective against bacteria	96.8	98.3	0.5
2. Antibiotics are effective against viruses	92.8	86.2	0.1
3. Antibiotics kill both the beneficial and non-beneficial bacteria	87.3	79.7	0.1
4. Colds are caused by bacteria	79.4	74.6	0.4
5. Colds are caused by viruses	89.1	89.7	0.9
6. Antibiotics speed up recovery from a cold	89.6	79.7	**0.04**
7. The majority of mild infections get better on their own	81.4	70.0	0.05
*Antibiotic resistance*			
8. Humans can be resistant to antibiotics	22.4	23.3	0.9
9. Bacteria can be resistant to antibiotics	97.2	91.5	**0.04**
10. Unnecessary use of an antibiotic can increase the resistance of bacteria to them	94.0	81.4	**<0.01**
11. Resistance can spread from human to human	72.5	67.2	0.4
12. Resistance is a growing problem in Denmark	85.7	66.1	**<0.01**
13. Resistance is only a concern for people who regularly use antibiotics	46.0	41.7	0.5
14. Resistance can eventually lead to incurable infections	94.8	81.4	**<0.01**

Bold numbers indicate p-values that are significant on the 95% confidence limit.

*n* = 312

The difference in correct responses was statistically significant in 5 of the 14 statements (*P* < 0.05). A higher percentage of native HCWs responded correctly to the statements compared to the percentage of foreign HCWs.

#### Knowledge-of-antibiotic score at baseline and after educational seminar

Totally, 307 observations at baseline had complete information on demographics and knowledge score. And 226 of baseline participants responded to the follow-up questionnaire resulting in a loss to follow-up rate of 32% from baseline to follow-up. At follow-up, 6 observations were excluded due to missing demographic information and 22 were excluded due to responses to too few statements. Therefore, the total number of observations included in the linear mixed model analyses included 307 at baseline and 198 at follow-up.

Table 3 presents the linear mixed model regression of the association between ethnicity and knowledge-of-antibiotic score. A significant negative correlation was found between respondents’ knowledge-of-antibiotic score and their ethnicity at baseline (−5.64, *P* = 0.004), signifying that native HCWs were more likely to have a better knowledge of antibiotics compared to foreign HCWs also after adjusting for work experience, place of employment, and educational background. The average proportion of statements responded correctly improved throughout the study period for both native HCWs (4.87) and foreign HCWs (5.78). There was no difference in the change in knowledge-of-antibiotic score after the intervention between native and foreign HCWs (0.91, *P* = 0.701).

**Table 3.** Results from linear mixed model regression assessing baseline knowledge and changes in knowledge score after the educational seminar by foreign and native healthcare workers

		Coefficient (95% CI)	*P*	Coefficient (95% CI)*	*P*
Intercept		80.50 (78.79;82.22)		79.09 (73.95;84.24)	
Ethnicity					
	Natives	Ref		Ref	
	Foreign	−7.53 (−11.45;−3.61)	<0.001	−5.64 (−9.46;−1.81)	0.004
Intervention					
	Pretest	Ref		Ref	
	Posttest	5.20 (3.15;7.26)	<0.001	4.87 (2.83;6.92)	<0.001
Intervention*Ethnicity					
	Natives at pretest	Ref		Ref	
	Foreign at pretest	Ref		Ref	
	Native at posttest	Ref		Ref	
	Foreign at posttest	0.51 (−4.20;5.23)	0.828	0.91 (−3.77;5.59)	0.701

* Adjusted for work experience and education. Place of employment included as random effect.

## Discussion

This study examines the relationship between knowledge of antibiotics and ethnicity among HCWs employed at nursing homes. Our findings suggest that differences exist in the knowledge of antibiotics among HCWs dependent of the country of birth. HCWs born in another country than Denmark have less baseline knowledge of antibiotics compared to HCWs born in Denmark. This association cannot be explained by ethnic differences in work experience, place of employment, or education alone.

Several studies have examined the level of knowledge of antibiotics or related subjects among parents, children, the public, nurses, or general practitioners (Alzoubi *et al*., 2009; André *et al*., 2010; Dyar *et al*., 2014; McCullough *et al*., 2016; Price *et al*., 2018). Notably, none of the identified studies have included HCWs as their target group or examined the level of knowledge about antibiotics among employees in nursing homes. Only a few studies have considered ethnicity as explaining determinant (Vanden Eng *et al*., 2003; Norris *et al*., 2010). Abera *et al*. (2014) and Alzoubi *et al*. (2009) examined knowledge and beliefs on antimicrobial resistance among physicians, pharmacists, and nurses (Alzoubi *et al*., 2009; Abera *et al*., 2014). Their findings indicated that most healthcare professionals were not up to date with knowledge on antimicrobial resistance. When it comes to providing healthcare professionals in the nursing home system with an adequate level of knowledge of antibiotics, our findings support that current education, in general, is insufficient.

The HCWs’ knowledge of antibiotics may be predefined by cultural identity, in this study defined by country of birth. It could be hypothesized that if the educational curriculum about bacteria, virus, and antibiotics had a compelling effect, differences in knowledge of antibiotics between native HCWs and foreign HCWs would disappear. This hypothesis disregards the effect of individuals’ cultural identity and/or assumes that aspects of culture can purposely be changed. Hofstede’s model of cultural dimensions has been stressed when researchers wanted to explain which cultural dimensions affect the perception of illness and the use of antibiotic (Deschepper *et al*., 2008; Gould and Meer, 2008; Hofstede, 2011; Gaygısız *et al*., 2017). The model describes how different values, traditions, and beliefs can partly explain the geographical differences in the use of antibiotics around the world (Brauer *et al*., 2016; ECDC, 2017). The findings in our study support this theory, which suggest that patterns of illness-related thinking founded by social background, that is, cultural dimensions, cannot easily be replaced with different views introduced in educational/professional settings.

For several reasons, no conclusions can be drawn in the study about the causal relationship between ethnicity and knowledge of antibiotics. As we have no measure of cultural identity, this also applies to any association between cultural identity and the beliefs about the appropriate use of antibiotics. The applied country of birth variable is only a proxy measure for cultural identity and we have no direct knowledge of the participants’ cultural identity. If we believed that in the country of birth was a proper proxy measure, we would assume that one’s country of birth has an impact on one’s behavior and thinking. An individual could be born in another country and move to Denmark with his/her Danish parents as a baby, then the country of birth would assumingly not affect the individuals’ cultural identity. The opposite case also applies. Being born in Denmark and having foreign parents does not necessarily imply a Danish cultural identity. Cultural identity is a multifaceted variable, and an overall idea covering a different set of norms, behavior, beliefs, and values. Ethnic categories such as language and country of birth can be used to represent elements of cultural identity but not define it (Bradby, 2003), which is why studies with more sophisticated measures of cultural identity should explore the relationship further.

Adjustment of covariates is often based on observed baseline imbalance between the compared groups, in this case, native HCWs and foreign HCWs. We found no significant imbalance in the educational background between the groups but chose deliberately to include the variable in the adjustment set anyway as omitting variables based solely on statistical balance from the model can result in bias (Groenwold *et al*., 2011; Lee, 2015). The adjustment set is thus decided on the theoretical basis that education and work experience potentially alter HCWs’ cultural identity in terms of knowledge, belief about antibiotics.

This study has several limitations. Native Danish was used in both the applied questionnaires and the seminar. This could be a potential source of bias, especially for foreign HCWs, as language barriers could lead to wrong responses or affect their level to advance. This problem could potentially strengthen the association of being a foreign HCW and having (false) limited knowledge of antibiotics, ultimately leading to a type 2 error. This is not considered a major issue as the participants’ Danish language proficiency is believed to be good, since the majority has received a Danish education.

In the construction of the binary variable: native HCWs versus foreign HCWs, we simplify the concept of ethnicity and ignore cultural variation between each country. The limited size of the dataset made it impossible to subdivide the participants into smaller geographical groups and hereby clarify if some countries distinguish themselves significantly from other countries in the knowledge of antibiotics. Our findings are based on analyses of a very heterogeneous group with many nationalities and cultures grouped. There are reasons to believe that variations exist in health belief between countries close to each other (Deschepper *et al*., 2008; Gaygısız *et al*., 2017). Detailed information would be beneficial in the process of designing intervention or educational curriculum targeting certain ethnic groups among HCWs. All analyses were performed without participants belonging to the educational group ‘Other’, as the impact on antibiotic prescribing from these workers are likely to be minimal. This changed the result in relation to differences in baseline knowledge of antibiotics marginally, leading to an insignificant correlation. This indicate that the dataset is deficient and the margin of error is big. This calls for studies that replicate our study but on a larger sample. Nineteen participants were excluded due to item nonresponse. Excluding participants on this basis could potentially lead to two problems. The most obvious drawback is the loss of statistical power. The second drawback is the risk of introducing bias. A higher percentage of foreign HCWs was represented in the excluded group when compared with the percentage of foreign HCWs in the analyzed sample. This could result in an underestimated coefficient if item nonresponse indicates a lack of knowledge. We choose deliberately not to perform imputation, as the number of item nonresponse is considered negligible. This study’s seven research locations were all within two municipalities. In the Capital Region of Denmark, public nursing homes follow municipality regulations that limit the generalizability across municipalities.

Another important limitation is the absence of a comparison group when we evaluate the effect of the educational seminar. Without a comparison group, we cannot attribute the increase in knowledge to the educational seminar. This inability to attribute causality is of major importance. The increase in knowledge level can be due to other reason, for example, local campaign with a general focus on decreasing unnecessary use of antibiotics. We also find it important to address that the differences in knowledge-of-antibiotic score found between native and foreign HCWs are negligible although significant. We would not expect such a difference was identifiable in an everyday setting, thus not clinically important. The same argument applies when discussing the changes in knowledge and awareness scores by foreign and native HCWs. The differences are very small. The questionnaire used in the present study is short, and one can only speculate if the differences were greater if we had introduced a large in-depth questionnaire.

One of the strengths of this study is the baseline response rate of 71%. This response rate seems to be a reasonable response rate for a paper-based survey when reviewing other studies’ response rate (Nulty, 2008). Another obvious strength is the originality of the study. We have not been able to identify any studies that have examined ethnic variations in knowledge of antibiotics among HCWs, although this subject may have significant impact on antibiotic use.

In Denmark, the link between the nursing home resident and the nursing home resident’s general practitioner who prescribes antibiotics is the HCW. Therefore, a key to reducing inappropriate use of antibiotics among nursing home residents in Denmark is to understand their knowledge of antibiotics. We need to understand the HCWs’ background and acknowledge cultural differences in antibiotic perspective to advance their knowledge of appropriate use of antibiotic. Previous studies have shown that healthcare providers at nursing home need more education about antibiotics and asymptomatic bacteriuria (Walker *et al*., 2000). Besides, there has been an increasing effort to recruit ethnic HCWs into nursing homes in Denmark (*Arbejdsvilkår i ældreplejen*, 2016). Consequently, a greater number of ethnic individuals have pursued a healthcare education in Denmark in the past years (Borg *et al*., 2005). The fact that HCWs, in general, need supplementary education about antibiotics along with the fact that students with diverse cultural basis and belief about antibiotics represents a greater percentage in health education institutions makes this issue important, especially if the inappropriate use of antibiotics in a nursing home should be reduced.

## Conclusions

The present study showed that differences exist in knowledge and awareness of appropriate use of antibiotics among HCWs in Danish nursing homes dependent on the country of birth. Our findings indicate that HCWs born outside Denmark reveal poorer knowledge and lower awareness concerning bacteria, viruses, antibiotics, and antibiotic resistance despite work experience, place of employment, and educational background. An educational seminar increased both native and foreign HCWs’ knowledge of antibiotics from baseline to follow-up, but differences in knowledge between native and foreign HCWs were maintained. Future interventions or educational efforts targeting HCWs should consider and understand the recipient’s ethnic origin. The findings should be investigated further in studies with larger samples size and more emphasis on cultural beliefs. Future studies should also include a more detailed questionnaire or more precise measures to evaluate knowledge of appropriate use of antibiotics, and we encourage intervention studies to put more effort into an intervention that entails reinforcement training.
